# Supplementary material for: CMR-net: A cross modality reconstruction network for multi-modality remote sensing classification
Source: PLoS One. 2024 Jun 25;19(6):e0304999. doi: 10.1371/journal.pone.0304999 (PMC11198768; doi:10.1371/journal.pone.0304999)
Supplement: Attachment — Submitted filename: Response to Reviewers.docx [file pone.0304999.s001.docx]

Dear Editors:

We would like to submit the enclosed manuscript entitled “CMR-Net: A Cross Modality Reconstruction Network for Multi-modality Remote Sensing Classification”, which we wish to be considered for publication in “PLOS ONE”. Regarding the reference to "Did you receive any third party support in conducting this research, analyzing the data, or preparing the manuscript for submission? If yes, provide details as to the organization(s) involved and their specific contributions."

The response was as follows: There was no third-party support in conducting this research, analyzing the data, or preparing the manuscript for submission.

Thank you and best regards.
Yours sincerely,
Huiqing Wang
Corresponding author:
Name: Huiqing Wang
E-mail: whq@swmu.edu.cn
