# Supplementary material for: CMR-net: A cross modality reconstruction network for multi-modality remote sensing classification
Source: PLoS One. 2024 Jun 25;19(6):e0304999. doi: 10.1371/journal.pone.0304999 (PMC11198768; doi:10.1371/journal.pone.0304999)
Supplement: Attachment — Submitted filename: Review Comments_Sep10.docx [file pone.0304999.s002.docx]

**Review Comments**

Thank you for the opportunity to peer-review this paper. The paper introduces a convolutional neural network (CNN)-based architecture called CMR-Net for the classification of multimodal remote sensing imagery data. The CMR-Net incorporates a Cross Modality Reconstruction (CMR) module for feature fusion, which the authors claim offers superior advantages in terms of classification results and performance compared to traditional fusion techniques.

The following observations have been raised;

1. The paragraphs are not numbered, which should be done so comments and suggestions can be pointed out easily.
2. Chapter arrangements, numbering and formatting should be corrected in line with this journal’s recommended formatting guidelines.
3. The paper does not discuss any potential drawbacks or shortcomings of the CMR-Net method in comparison to other state-of-the-art algorithms. Hence, the authors should provide a detailed discussion on the limitations and challenges faced during the implementation of the CMR-Net architecture, as this would help in understanding the potential drawbacks of the proposed method.
4. Conduct a comparative analysis with a wider range of state-of-the-art algorithms for multi-modality remote sensing data classification, to provide a more comprehensive evaluation of the performance of CMR-Net.
5. Include a discussion on the computational cost and efficiency of the CMR-Net method, as increasing the number of layers in the network may impact the convergence speed and computational requirements.
