# Supplementary material for: CMR-net: A cross modality reconstruction network for multi-modality remote sensing classification
Source: PLoS One. 2024 Jun 25;19(6):e0304999. doi: 10.1371/journal.pone.0304999 (PMC11198768; doi:10.1371/journal.pone.0304999)
Supplement: Attachment — Submitted filename: Review Report- PONE-D-23-24147.docx [file pone.0304999.s003.docx]

The article is technically sound and presented in appropriate manner. Also, the title reflects the contents of the paper. However, the author(s) are suggested to include the proposed modifications given as follows-

1. The introductory section does not adequately explain the framework and problems of research in the respective area. Elaborate the introduction part and state the motivation clearly.
2. Give the rationale of the study that justifies why the specific approach has been used by the author(s).
3. The review of literature section is merged with the introduction part. It should be written under a separate heading ‘Related Work’. Also, the author(s) are advised to add a few latest studies relevant to the area in this section.
4. The clarity of presentation of mathematical foundation of the method is weak. Elaborate the same and the variables used in the equations should be well denoted and explained.
5. The results are not suitably described. It is suggested to put in the details and strengthen the same.
6. After the result section, add a paragraph to clearly explain how your study represents incremental advance over previously published work.
7. The limitations of the work done should be discussed in the conclusion section. Discuss the major limitations of the study.
8. Linguistic/grammatical/typographical error should be corrected before publication.
