# Supplementary material for: CMR-net: A cross modality reconstruction network for multi-modality remote sensing classification
Source: PLoS One. 2024 Jun 25;19(6):e0304999. doi: 10.1371/journal.pone.0304999 (PMC11198768; doi:10.1371/journal.pone.0304999)
Supplement: Attachment — Submitted filename: Response to Reviewers.docx [file pone.0304999.s004.docx]

**Response Comments**

I would like to thank the reviewers for reviewing CMR-Net: A Cross Modality Reconstruction Network for Multi-modality Remote Sensing Classification, and I sincerely accept the suggestions made, and I have made the corresponding modifications, which will help me a lot in my later research. This will help me a lot in my later research. I would like to respond to the suggestions as follows:

1. The introductory section does not adequately explain the framework and problems of research in the respective area. Elaborate the introduction part and state the motivation clearly.

**Response:** Thank you very much for the review suggestions, which are very helpful for my later research work. I have followed the suggestions in the "Introduction" and "Related work" sections of the article, respectively, from the traditional methods of multimodal remote sensing classification based on morphological and subspace classification methods, and then to the current deep learning methods in various fields of research frameworks. The research frameworks of various fields from the traditional methods of multimodal remote sensing classification based on morphology and subspace, and then to the current deep learning methods are analysed and explained.

2. Give the rationale of the study that justifies why the specific approach has been used by the author(s).

**Response:** First of all, I would like to thank you very much for the review suggestions, which are very helpful for my later research work. I have described the background and principle of this research and proposed the specific research methodology in the "Introduction" part of the article according to the suggestions, and then described the current status of the related research in the "Related work" part according to the temporal clues of the development of multimodal remote sensing data categorization.

3. The review of literature section is merged with the introduction part. It should be written under a separate heading ‘Related Work’. Also, the author(s) are advised to add a few latest studies relevant to the area in this section.

**Response:** First of all, I would like to thank you very much for the review suggestions, which are very helpful for my later research work. According to the suggestion, I have merged the literature review with the introduction, added the section of "Related work", and added the latest research in this field in 2022 in the literature.

4. The clarity of presentation of mathematical foundation of the method is weak. Elaborate the same and the variables used in the equations should be well denoted and explained.

**Response:** First of all, I would like to thank you very much for the review suggestions, which are very helpful for my later research work. According to the suggestion, I have clearly revised the mathematical formulas and foundations in the "Materials and methods" part of the article to make them clear, and added detailed descriptions and illustrations of the formulas.

5. The results are not suitably described. It is suggested to put in the details and strengthen the same.

**Response:** First of all, I would like to thank very much for the review suggestions, which are very helpful for my later research work. I have followed the suggestion to add a "Result and discussion" section to the article, which is used to describe the related work of this study, and at the same time, I have improved and modified the description of the experimental results and analysis in the "Experimental setup", and increased the details of the presentation.

6. After the result section, add a paragraph to clearly explain how your study represents incremental advance over previously published work.

**Response**: First of all, I would like to thank you very much for the review suggestions, which are very helpful for my later research work. As suggested, I have added a "Results and discussion" section after the results section in the article to explain that the present study is based on previously published research work and is an improvement of the work based on previous research.

7. The limitations of the work done should be discussed in the conclusion section. Discuss the major limitations of the study.

**Response**: First of all, I am very thankful for the review suggestions given, which will be very helpful for my future research work. As per the suggestions, I have discussed the limitations of the research work in this paper in the conclusion section of the article. That is, "This paper also faces some limitations and difficulties in the process of research work, mainly due to the fact that the multimodal remote sensing data sources are relatively few and difficult to obtain, and most of them are small samples of data, which is a limitation for a larger research work. Meanwhile, the proposed CMR-Net method involves some parameter tuning, which requires a lot of computation time during the training process and a better performing GPU card, which limits the fast presentation of the computational results."

8. Linguistic/grammatical/typographical error should be corrected before publication.

**Response**: First of all, I am very grateful for the advice given, I have scrutinized the article for some grammatical and typographical errors, and the problems found have been revised, and will be completed at a later stage before publication as required.

9. The paragraphs are not numbered, which should be done so comments and suggestions can be pointed out easily.

**Response**: Thank you very much for suggesting the review, it will be very helpful for my later research. Based on the suggestions I have revised and reformatted the paper according to the recommended formatting guidelines of the journal.

10. Chapter arrangements, numbering and formatting should be corrected in line with this journal’s recommended formatting guidelines.

**Response**: Suggestions for review are greatly appreciated and have been revised and reformatted in accordance with the journal's recommended formatting guidelines.

11. The paper does not discuss any potential drawbacks or shortcomings of the CMR-Net method in comparison to other state-of-the-art algorithms. Hence, the authors should provide a detailed discussion on the limitations and challenges faced during the implementation of the CMR-Net architecture, as this would help in understanding the potential drawbacks of the proposed method.

**Response:** Thank you very much for the review suggestion, which is very helpful for my later research work. I have suggested that the limitations and challenges faced during the implementation of the CMR-Net method are discussed in detail in the "Result and discussion" section of the article, and also pointed out that the method increases the number of CNN convolutional layers and spends more computational overheads and does not provide the best results. It is also pointed out that the method increases the number of layers of CNN convolutional layers and costs more computational overhead and is not validated by fusing data from more data sources (e.g., three data sources).

12. Conduct a comparative analysis with a wider range of state-of-the-art algorithms for multi-modality remote sensing data classification, to provide a more comprehensive evaluation of the performance of CMR-Net.

**Response:** Thank you very much for the review suggestions, for my later research work is very helpful, I have been in accordance with the suggestions in the article in the "Experimental setup" part of the comparative analysis with the state-of-the-art algorithms in the introduction of the latest literature put forward EndNet method, as well as other deep learning methods for performance comparison, classification accuracy comparison and model training. The performance comparison, classification accuracy comparison and model training overhead are analyzed.

13. Include a discussion on the computational cost and efficiency of the CMR-Net method, as increasing the number of layers in the network may impact the convergence speed and computational requirements.

**Response:** Thank you very much for the review suggestions, which are very helpful for my later research work. I have discussed the computational cost and efficiency of the CMR-Net method due to the increase of the number of layers in the network in the "Result and discussion" part of the article according to the suggestions, which pointed out that in the implementation of the CMR-Net classification method, the number of layers in the CNN convolutional layer is increased and more computational overhead is spent. It is discussed that the CMR-Net classification method is implemented by increasing the number of CNN convolutional layers and incurring more computational overhead in order to extract multimodal features more adequately, and the method performs cross-modal feature fusion on the extracted features. It is more accurate compared to the other two deep learning methods, but it also increases the computational cost and the network convergence will be slower. This method of cross-modal feature fusion of the extracted features also increases the computational cost and the network convergence will be slower compared to the other two deep learning methods in the paper. It is observed experimentally that the computational overhead of CMR-Net is about 10 minutes more than EndNet. However, this can be changed by improving the performance of the GPU compute card.
