# Supplementary material for: CMR-net: A cross modality reconstruction network for multi-modality remote sensing classification
Source: PLoS One. 2024 Jun 25;19(6):e0304999. doi: 10.1371/journal.pone.0304999 (PMC11198768; doi:10.1371/journal.pone.0304999)
Supplement: Attachment — Submitted filename: Response to Reviewers.docx [file pone.0304999.s005.docx]

**Response Comments**

I would like to thank the academic editor and reviewers for reviewing CMR-Net: A Cross Modality Reconstruction Network for Multi-modality Remote Sensing Classification, and I sincerely accept the suggestions made, and I have made the corresponding modifications, which will help me a lot in my later research. This will help me a lot in my later research. I would like to respond to the suggestions as follows:

1. In the start of Introduction section: "Hyperspectral image (HS), multispectral image (MS)" , It is better to write as: Hyperspectral (HS) image, multispectral (MS) image.

**Response:** Thanks to the editor and reviewers for their comments, I have changed "Hyperspectral image (HS), multispectral image (MS)" to "Hyperspectral (HS) image, multispectral (MS) image" in the introduction. ".

2. "With the advancement of aerospace technology, Remote Sensing (RS) has emerged as a pivotal tool in Earth observation."     It is better to replace "Remote Sensing (RS)" with remote sensing (RS). and afterward use RS instead of the full term, Check the same in the whole manuscript to make it standard.

**Response:** Thanks to the comments of the editor and reviewers, I have double-checked the standardisation of "Remote Sensing (RS)" throughout the article and used the term "RS" instead of "remote sensing" in the article.

3. Same issue in "Index Terms-Classification, convolutional neural networks (CNNs), remote sensing (RS), Cross Modality Reconstruction (CMR), deep learning (DL), light detection and ranging (LiDAR), synthetic aperture radar (SAR)." It is better to keep the standard starting words with small letters and replace with;   cross modality reconstruction (CMR).

**Response:** Thanks to the comments made by the editor and reviewers, I have checked and modified the initial letters of the words in the index terms to lower case letters. The modification is: "Index Terms-classification, convolutional neural networks (CNN), remote sensing, cross modality reconstruction (CMR), deep learning (DL), light detection and ranging (LiDAR), synthetic aperture radar (SAR) light detection and ranging (LiDAR), synthetic aperture radar (SAR).".

4. Subheading of Results and discussion, "Data Description"  It is better to write as Data description.

**Response:** Thanks to the editor and reviewers for their comments, I have changed the subtitle of Results and Discussion from "Data Description" to "Data description".

5. Replace "berlin" with Berlin and make it correct.

**Response:** Thanks to the editor and the reviewers for their comments, I have checked and corrected "berlin" to "Berlin" in the article.

6. It is recommended to cite few relevant references of year 2023 and 2024.

**Response:** Thanks to the suggestions from the editors and reviewers, I have cited relevant literature from 2023 and 2024 in the "Related work" of the article, and modified the order and format of some of the literature in the "References". The references cited in 2023 and 2024 are as follows:

[30] F.M. Guo, Z.W. Li, et al, "Semi-supervised cross-domain feature fusion classification network for coastal wetland classification with hyperspectral and LiDAR data," INT J APPL EARTH OBS, vol. 120, 2023.

[31] Y.N. Feng, J.H. Zhang, et al. "S2EFT: Spectral-Spatial-Elevation Fusion Transformer for hyperspectral image and LiDAR classification," KNOWL-BASED SYST, vol. 283, 2024.

[33] 33. Xin. He, Y.S. Chen, et al, "Foundation Model-Based Multimodal Remote Sensing Data Classification. " IEEE Trans. Geosci. Remote Sens. vol. 62, 2024.
